# Supplementary material for: Economic and environmental competitiveness of multiple hydrogen production pathways in China
Source: Nat Commun. 2025 May 8;16:4284. doi: 10.1038/s41467-025-59412-y (PMC12062354; doi:10.1038/s41467-025-59412-y)
Supplement: Supplementary file 1 — Supplementary Information [file 41467_2025_59412_MOESM1_ESM.pdf]

Supplementary Information

Economic and environmental competitiveness of multiple hydrogen production pathways in China

Guangyao Fan[1], Hui Zhang[1], Bo Sun[1*], Fengwen Pan[2,3*]

[1]*School of Control Science and Engineering, Shandong University, Jinan, Shandong 250061, China*

[2]*Weichai Power Co., Ltd., Weifang 261061, China*

[3]*National Center of Technology Innovation for Fuel Cell, Weifang 26100, China*

**Corresponding authors.*

E-mail: sunbo@sdu.edu.cn (B. Sun) panfengwen@outlook.com (F. Pan)

Supplementary Note 1. Optimization model of water electrolysis hydrogen production systems

System 1: the grid hydrogen production system

Optimization objective

$$LCOH_{\mathrm{WE1}} = \frac{C_{\mathrm{inv,WE1}} + C_{\mathrm{om,WE1}} + C_{\mathrm{grid,WE1}} + C_{\mathrm{c,WE1}}}{\sum_{n=1}^{N} \frac{H_{\mathrm{output}}}{(1+r)^n}} \tag{1}$$

$$C_{\mathrm{inv,WE1}} = c_{\mathrm{inv,EL}} \cdot IC_{\mathrm{EL}} \tag{2}$$

$$C_{\mathrm{om,WE1}} = \sum_{n=1}^{N} \frac{c_{\mathrm{om,EL}} \cdot IC_{\mathrm{EL}}}{(1+r)^n} \tag{3}$$

$$C_{\mathrm{grid,WE3}} = \sum_{n=1}^{N} \frac{\sum_{t=1}^{t=h} \left(c_{\mathrm{grid}} \cdot EP_{\mathrm{grid}}(t) \right) + c_{\mathrm{grid,fix1}} \cdot EP_{\mathrm{grid,max}} + \frac{c_{\mathrm{grid,fix2}} \cdot EP_{\mathrm{grid,max}}}{\cos\varphi}}{(1+r)^n} \tag{4}$$

$$C_{\mathrm{c,WE1}} = c_{\mathrm{c}} \cdot LCCE_{\mathrm{WE1}} \tag{5}$$

Decision variables

The decision variable of configuration is the capacity of electrolyzer (IC_{EL}, kW). The decision variables of operation include the input power of the electrolyzer ($EP_{\mathrm{EL}}(t)$, kW), the production of hydrogen of the electrolyzer ($H_{\mathrm{EL}}(t)$, kg), the power purchased from the grid ($EP_{\mathrm{grid}}(t)$, kW).

Constraints

(a) Equipment operation constraints

$$H_{\mathrm{EL}}(t) \cdot LHV = \eta_{\mathrm{EL}}(t) \cdot EP_{\mathrm{EL}}(t) \tag{6}$$

$$0 \le EP_{\mathrm{EL}}(t) \le IC_{\mathrm{EL}} \tag{7}$$

$$0 \le IC_{\mathrm{EL}} \tag{8}$$

(b) Power purchase constraints

$$0 \le EP_{\mathrm{grid}}(t) \le EP_{\mathrm{grid,max}} \tag{9}$$

(c) Electricity balance constraints

$$EP_{\mathrm{grid}}(t) = EP_{\mathrm{EL}}(t) \tag{10}$$

(d) Hydrogen supply reliability constraints

$$H_{\mathrm{EL}}(t) = \frac{H_{\mathrm{output}}}{h} \tag{11}$$

System 2: the hydrogen production system combined photovoltaic and grid
Optimization objective

$$LCOH_{\mathrm{WE2}} = \frac{C_{\mathrm{inv,WE2}} + C_{\mathrm{om,WE2}} + C_{\mathrm{grid,WE2}} + C_{\mathrm{c,WE2}}}{\sum_{n=1}^{N} \frac{H_{\mathrm{output}}}{(1+r)^n}} \tag{12}$$

$$C_{\mathrm{inv,WE2}} = c_{\mathrm{inv,PV}} \cdot IC_{\mathrm{PV}} + c_{\mathrm{inv,EL}} \cdot IC_{\mathrm{EL}} \tag{13}$$

$$C_{\mathrm{om,WE2}} = \sum_{n=1}^{N} \frac{c_{\mathrm{om,PV}} \cdot IC_{\mathrm{PV}} + c_{\mathrm{om,EL}} \cdot IC_{\mathrm{EL}}}{(1+r)^n} \tag{14}$$

$$C_{\mathrm{grid,WE3}} = \sum_{n=1}^{N} \frac{\sum_{t=1}^{t=h} \left(c_{\mathrm{grid}} \cdot EP_{\mathrm{grid}}(t)\right) + c_{\mathrm{grid,fix1}} \cdot EP_{\mathrm{grid,max}} + \frac{c_{\mathrm{grid,fix2}} \cdot EP_{\mathrm{grid,max}}}{\cos\varphi}}{(1+r)^n} \tag{15}$$

$$C_{\mathrm{c,WE1}} = c_{\mathrm{c}} \cdot LCCE_{\mathrm{WE2}} \tag{16}$$

Decision variables

The decision variables of configuration are the capacity of photovoltaic panels (IC_{PV}, kW) and the capacity of electrolyzer (IC_{EL}, kW). The decision variables of operation include the input power of the electrolyzer ($EP_{\mathrm{EL}}(t)$, kW), the production of hydrogen of the electrolyzer ($H_{\mathrm{EL}}(t)$, kg), the power purchased from the grid ($EP_{\mathrm{grid}}(t)$, kW).

Constraints

(a) Equipment operation constraints

$$EP_{\mathrm{PV}}(t) = IC_{\mathrm{PV}} \cdot EP_{\mathrm{PV,0}}(t) \tag{17}$$

$$0 \le IC_{\mathrm{PV}} \tag{18}$$

$$H_{\mathrm{EL}}(t) \cdot LHV = \eta_{\mathrm{EL}}(t) \cdot EP_{\mathrm{EL}}(t) \tag{19}$$

$$0 \le EP_{\mathrm{EL}}(t) \le IC_{\mathrm{EL}} \tag{20}$$

$$0 \le IC_{\mathrm{EL}} \tag{21}$$

(b) Power purchase constraints

$$0 \le EP_{\mathrm{grid}}(t) \le EP_{\mathrm{grid,max}} \tag{22}$$

(c) Electricity balance constraints

$$EP_{\mathrm{PV}}(t) + EP_{\mathrm{grid}}(t) = EP_{\mathrm{EL}}(t) \tag{23}$$

(d) Hydrogen supply reliability constraints

$$H_{\mathrm{EL}}(t) = \frac{H_{\mathrm{output}}}{h} \tag{24}$$

System 4：the off-grid hydrogen production system

Optimization objective

$$LCOH_{\mathrm{WE4}} = \frac{C_{\mathrm{inv,WE4}} + C_{\mathrm{om,WE4}} + C_{\mathrm{c,WE4}}}{\sum_{n=1}^{N} \frac{H_{\mathrm{output}}}{(1+r)^n}} \tag{25}$$

$$C_{\mathrm{inv,WE4}} = c_{\mathrm{inv,PV}} \cdot IC_{\mathrm{PV}} + c_{\mathrm{inv,EL}} \cdot IC_{\mathrm{EL}} + c_{\mathrm{inv,EES}} \cdot IC_{\mathrm{EES}} + c_{\mathrm{inv,HST}} \cdot IC_{\mathrm{HST}} + c_{\mathrm{inv,com}} \cdot IC_{\mathrm{com}} \tag{26}$$

$$C_{\mathrm{om,WE4}} = \sum_{n=1}^{N} \frac{c_{\mathrm{om,PV}} \cdot IC_{\mathrm{PV}} + c_{\mathrm{om,EL}} \cdot IC_{\mathrm{EL}} + c_{\mathrm{om,EES}} \cdot IC_{\mathrm{EES}} + c_{\mathrm{om,HST}} \cdot IC_{\mathrm{HST}} + c_{\mathrm{om,com}} \cdot IC_{\mathrm{com}}}{(1+r)^n} \tag{27}$$

$$C_{\mathrm{c,WE4}} = c_{\mathrm{c}} \cdot LCCE_{\mathrm{WE4}} \tag{28}$$

Decision variables

The decision variables of configuration include the capacity of photovoltaic panels (IC_{PV}, kW), the capacity of electrolyzer (IC_{EL}, kW), the capacity of compressor (IC_{com}, kW), and the capacity of electricity energy storage (IC_{EES}, kWh), the capacity of the hydrogen storage tank (IC_{HST}, kg). The decision variables of operation include the input power of the electrolyzer ($EP_{\mathrm{EL}}(t)$, kW), the production of hydrogen of the electrolyzer ($H_{\mathrm{EL}}(t)$, kg), the power consumption of the compressor ($EP_{\mathrm{com}}(t)$, kW), the charging power of the electricity energy storage ($EP_{\mathrm{EES,im}}(t)$, kW), the discharging power of the electricity energy storage ($EP_{\mathrm{EES,ex}}(t)$, kW), hydrogen storage of the hydrogen storage tank ($H_{\mathrm{HST,im}}$(t), kg), hydrogen release of the hydrogen storage tank ($H_{\mathrm{HST,ex}}$(t), kg).

Constraints

(a) Equipment operation constraints

$$EP_{\mathrm{PV}}(t) = IC_{\mathrm{PV}} \cdot EP_{\mathrm{PV,0}}(t) \tag{29}$$

$$0 \le IC_{\mathrm{PV}} \tag{30}$$

$$H_{\mathrm{EL}}(t) \cdot LHV = \eta_{\mathrm{EL}}(t) \cdot EP_{\mathrm{EL}}(t) \tag{31}$$

$$0 \le EP_{\mathrm{EL}}(t) \le IC_{\mathrm{EL}} \tag{32}$$

$$0 \le IC_{\mathrm{EL}} \tag{33}$$

$$EP_{\mathrm{com}}(t) = EP_{\mathrm{com,0}} \cdot H_{\mathrm{EL}}(t) \cdot LHV \tag{34}$$

$$EP_{\mathrm{com}}(t) \le IC_{\mathrm{com}} \tag{35}$$

$$\begin{cases} E_{\text{EES}}(t+1) = E_{\text{EES}}(t)(1-\alpha) + (EP_{\text{EES,im}}(t)\eta_{\text{EES,im}} - EP_{\text{EES,ex}}(t)/\eta_{\text{EES,ex}})\Delta t \\ SOC_{\text{EES}}(t) = E_{\text{EES}}(t)/IC_{\text{EES}} \\ SOC_{\text{EES,min}} \le SOC_{\text{EES}}(t) \le SOC_{\text{EES,max}} \\ 0 \le EP_{\text{EES,im}}(t) \le EP_{\text{EES,im,max}} \\ 0 \le EP_{\text{EES,ex}}(t) \le EP_{\text{EES,ex,max}} \\ EP_{\text{EES,im}}(t) \cdot EP_{\text{EES,ex}}(t) = 0 \\ SOC_{\text{EES}}(t_{\text{start}}) = SOC_{\text{EES}}(t_{\text{end}}) \end{cases} \tag{36}$$

$$\begin{cases} E_{\text{EES}}(t+1) = E_{\text{EES}}(t)(1-\alpha) + (EP_{\text{EES,im}}(t)\eta_{\text{EES,im}} - EP_{\text{EES,ex}}(t)/\eta_{\text{EES,ex}})\Delta t \\ SOC_{\text{EES}}(t) = E_{\text{EES}}(t)/IC_{\text{EES}} \\ SOC_{\text{EES,min}} \le SOC_{\text{EES}}(t) \le SOC_{\text{EES,max}} \\ 0 \le EP_{\text{EES,im}}(t) \le EP_{\text{EES,im,max}} \\ 0 \le EP_{\text{EES,ex}}(t) \le EP_{\text{EES,ex,max}} \\ EP_{\text{EES,im}}(t) \cdot EP_{\text{EES,ex}}(t) = 0 \\ SOC_{\text{EES}}(t_{\text{start}}) = SOC_{\text{EES}}(t_{\text{end}}) \end{cases} \tag{37}$$

(b) Electricity balance constraints

$$EP_{\text{PV}}(t) + EP_{\text{EES,ex}}(t) = EP_{\text{EES,im}}(t) + EP_{\text{EL}}(t) + EP_{\text{com}}(t) \tag{38}$$

(c) Hydrogen energy balance constraint

$$H_{\text{EL}}(t) = H_{\text{EL,out}}(t) + H_{\text{HST,im}}(t) \tag{39}$$

(d) Hydrogen supply reliability constraints

$$H_{\text{EL,out}}(t) + H_{\text{HST,ex}}(t) = \frac{H_{\text{output}}}{h} \tag{40}$$

Supplementary Note 2. Data and parameters of optimization model

Supplementary Table 1. Technical and economic parameters of water electrolysis hydrogen production systems.

Equipment	Parameter	Symbol	Value	Source
Photovoltaic panel	Investment cost of photovoltaic panel ($/kW)	$c_{\mathrm{inv,PV}}$	522	[1]
	Fixed operation and maintenance cost of photovoltaic panel ($/kW-year)	$c_{\mathrm{om,PV}}$	52.2	
Electrolyzer	Investment cost of electrolyzer (Taking PEM electrolyzer as an example, $/kW)	$c_{\mathrm{inv,EL}}$	1606.7	[2]
	Fixed operation and maintenance cost of electrolyzer ($/kW-year)	$c_{\mathrm{om,EL}}$	89.2	
	Rated conversion efficiency of electrolyzer (%)	η_{EL}	61.3	
Electricity energy storage	Investment cost of electricity energy storage ($/kWh)	$c_{\mathrm{inv,EES}}$	344.2	[3, 4, 5, 6]
	Fixed operation and maintenance cost of electricity energy storage ($/kWh-year)	$c_{\mathrm{om,EES}}$	8.6	
	Charging efficiency (%)	$\eta_{\mathrm{EES,im}}$	0.945	
	Discharging efficiency (%)	$\eta_{\mathrm{EES,ex}}$	0.945	
	The self-discharge rate (%/hour)	α	0.002	
	The maximum charging power (kW)	$EP_{\mathrm{EES,im,max}}$	$0.2\times IC_{\mathrm{EES}}$	
	The maximum discharging power (kW)	$EP_{\mathrm{EES,ex,max}}$	$0.2\times IC_{\mathrm{EES}}$	
	The minimum state of charge	$SOC_{\mathrm{EES,min}}$	0.2	
	The maximum state of charge	$SOC_{\mathrm{EES,max}}$	0.8	
Hydrogen storage tank	Investment cost of hydrogen storage tank ($/kg)	$c_{\mathrm{inv,EES}}$	816.3	[6, 7, 8]
	Fixed operation and maintenance cost of hydrogen storage tank ($/kg-year)	$c_{\mathrm{om,EES}}$	8.2	
	The maximum rates of hydrogen import	$H_{\mathrm{HST,im,max}}$	IC_{HST}	
	The maximum rates of hydrogen export	$H_{\mathrm{HST,ex,max}}$	IC_{HST}	
	The minimum level of hydrogen (%)	$LOH_{\mathrm{HST,min}}$	0	
	The maximum level of hydrogen (%)	$LOH_{\mathrm{HST,max}}$	100	
	Pressure of hydrogen storage tank (bar)	p_{HST}	200	
Compressor	Investment cost of compressor ($/kWh)	$c_{\mathrm{inv,com}}$	2666.3	[6]
	Fixed operation and maintenance cost of compressor ($/kWh-year)	$c_{\mathrm{om,com}}$	106.6	

General	System life (years)	N	20	[2, 9]
	Discount rate (%)	r	3.85	

Supplementary Table 2. Electricity prices of 31 provinces.

Province	Electricity price ($/kWh)	Capacitive demand electricity price	
		Maximum demand ($/kW-year)	Transformer capacity ($/kVA-year)
Beijing (BJ)	0.0853	79.3	52.9
Tianjin (TJ)	0.0957	42.1	28.1
Shanghai (SH)	0.1056	69.4	46.3
Chongqing (CQ)	0.0894	59.5	39.7
Liaoning (LN)	0.0751	54.5	36.4
Jilin (JL)	0.0868	54.5	36.4
Heilongjiang (HL)	0.0827	54.5	36.4
Inner Mongolia (IM)	0.0660	46.3	31.4
Shanxi (SX)	0.0679	59.5	39.7
Hebei (HE)	0.0875	57.9	38.3
Shandong (SD)	0.0941	62.8	46.3
Shaanxi (SN)	0.0770	51.2	36.4
Ningxia (NX)	0.0566	49.6	33.1
Gansu (GS)	0.0593	47.1	31.4
Qinghai (QH)	0.0519	47.1	31.4
Xinjiang (XJ)	0.0542	54.5	43.0
Tibet (XZ)	0.0930	47.1	31.4
Sichuan (SC)	0.0565	54.5	36.4
Yunnan (YN)	0.0614	61.2	44.6
Guangxi (GX)	0.0949	56.2	45.5
Guizhou (GZ)	0.0805	52.9	38.0
Hunan (HN)	0.0959	49.6	33.1
Guangdong (GD)	0.0956	52.9	38.0
Fujian (FJ)	0.0819	56.5	37.7
Zhejiang (ZJ)	0.0960	66.1	49.6
Jiangxi (JX)	0.0951	64.5	43.0
Anhui (AH)	0.0875	66.1	49.6
Jiangsu (JS)	0.0899	66.1	49.6
Hubei (HB)	0.0931	62.8	41.3
Henan (HA)	0.0956	46.3	33.1
Hainan (HI)	0.0863	62.8	43.0

Source: 35kV large-scale industrial electricity retail electricity price is the electricity price purchased from the power grid. The electricity price comes from the electricity price sales table of each provincial power grid company.

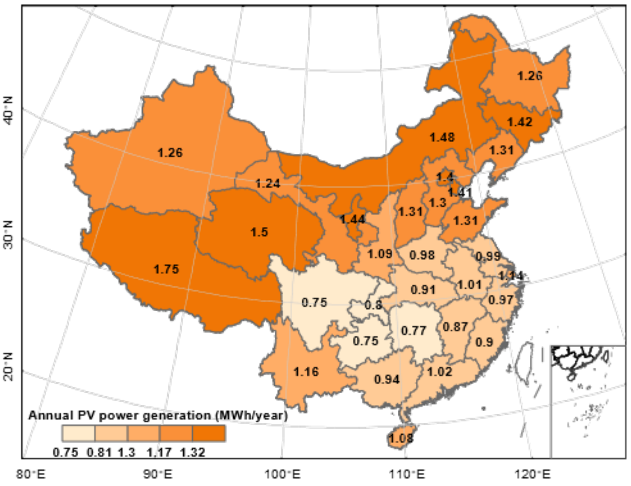

Supplementary Figure 1. Annual photovoltaic power generation in each province of China. The data of municipalities are directly obtained from PVWatts Calculator [10]. For different provinces, the average photovoltaic power generation data of the province is calculated in five cities distributed in different locations. For example, five representative cities, Jinan, Qingdao, Zaozhuang, Tai'an, and Weifang, are selected to calculate average photovoltaic power generation in Shandong province. Map Source: Standard Map Service of the Ministry of Natural Resources, People's Republic of China [Map Review Number: GS (2020) 4619].

Supplementary Table 3. Technical and economic parameters of hydrogen production from coal.

Equipment	Unit investment cost ($/kW H_2)	Annual operation and maintenance cost ($/kW H_2-year)	Efficiency (LHV)	Carbon emission coefficient (kg CO_2/kg H_2)
CG	2537.1 [2]	126.9 [2]	60 % [2]	22.65 [11]
CG+CCUS	2642.1 [2]	132.1 [2]	58 % [2]	10.59 [11]

Supplementary Table 4. Technical and economic parameters of hydrogen production from natural gas[3].

Equipment	Unit investment cost ($/kW H_2)	Annual fixed operation and maintenance cost ($/kW-year H_2)	Electricity Use (kWh/kg H_2)	Natural Gas Use (kg CH_4/kg H_2)	Direct carbon emission coefficient (kg CO_2/kg H_2)
SMR	539.9	25.6	0.65	3.53	9.3
SMR+CCUS	1313.9	57	2.04	3.75	0.4

Supplementary Table 5. Natural gas prices of 31 provinces.

Province	Natural gas price ($/$m^3$)
Beijing (BJ)	0.395
Tianjin (TJ)	0.489
Shanghai (SH)	0.503
Chongqing (CQ)	0.497
Liaoning (LN)	0.500
Jilin (JL)	0.421
Heilongjiang (HL)	0.536
Inner Mongolia (IM)	0.179
Shanxi (SX)	0.410
Hebei (HE)	0.554
Shandong (SD)	0.468
Shaanxi (SN)	0.481
Ningxia (NX)	0.463
Gansu (GS)	0.398
Qinghai (QH)	0.318
Xinjiang (XJ)	0.310
Tibet (XZ)	0.614
Sichuan (SC)	0.579
Yunnan (YN)	0.375
Guangxi (GX)	0.565
Guizhou (GZ)	0.512
Hunan (HN)	0.450
Guangdong (GD)	0.357
Fujian (FJ)	0.584
Zhejiang (ZJ)	0.579
Jiangxi (JX)	0.537
Anhui (AH)	0.573
Jiangsu (JS)	0.444
Hubei (HB)	0.511
Henan (HA)	0.650
Hainan (HI)	0.483

Source: https://www.ceicdata.com.cn/zh-hans/china/gas-price-36-city [12]

Supplementary Table 6. Cost and carbon emissions of hydrogen production from industrial by-product.

The type of industrial by-product hydrogen	LCOH ($/kg H_2)	LCCE (kg CO2/kg H_2)
Hydrogen by-production from light hydrocarbon cracking	1.9-2.8	8.9-12.9
Hydrogen by-production from Chlor-alkali	1.8-2.8	

Hydrogen by-production from Coke oven gas	1.3-2.0	
Hydrogen by-production from ammonia synthesis, methanol synthesis, etc.	2.0-3.1	
Average value	2.2	10.9

Source: Hydrogen Energy Industry Development Report of China 2020 [13]

Supplementary Table 7. Carbon emission factors of electricity purchase in 31 provinces.

Province	Carbon emission factor (kg CO_2/kWh)
Beijing (BJ)	0.615
Tianjin (TJ)	0.841
Shanghai (SH)	0.548
Chongqing (CQ)	0.432
Liaoning (LN)	0.91
Jilin (JL)	0.839
Heilongjiang (HL)	0.814
Inner Mongolia (IM)	1.000
Shanxi (SX)	0.841
Hebei (HE)	1.092
Shandong (SD)	0.742
Shaanxi (SN)	0.641
Ningxia (NX)	0.872
Gansu (GS)	0.46
Qinghai (QH)	0.095
Xinjiang (XJ)	0.749
Tibet (XZ)	0.095
Sichuan (SC)	0.117
Yunnan (YN)	0.146
Guangxi (GX)	0.526
Guizhou (GZ)	0.42
Hunan (HN)	0.487
Guangdong (GD)	0.445
Fujian (FJ)	0.489
Zhejiang (ZJ)	0.532
Jiangxi (JX)	0.616
Anhui (AH)	0.763
Jiangsu (JS)	0.695
Hubei (HB)	0.316
Henan (HA)	0.738
Hainan (HI)	0.459

Source: China regional power grids carbon dioxide emission factors (2023) [14]

Supplementary Note 3. Operation characteristics of water electrolysis hydrogen production systems in Beijing

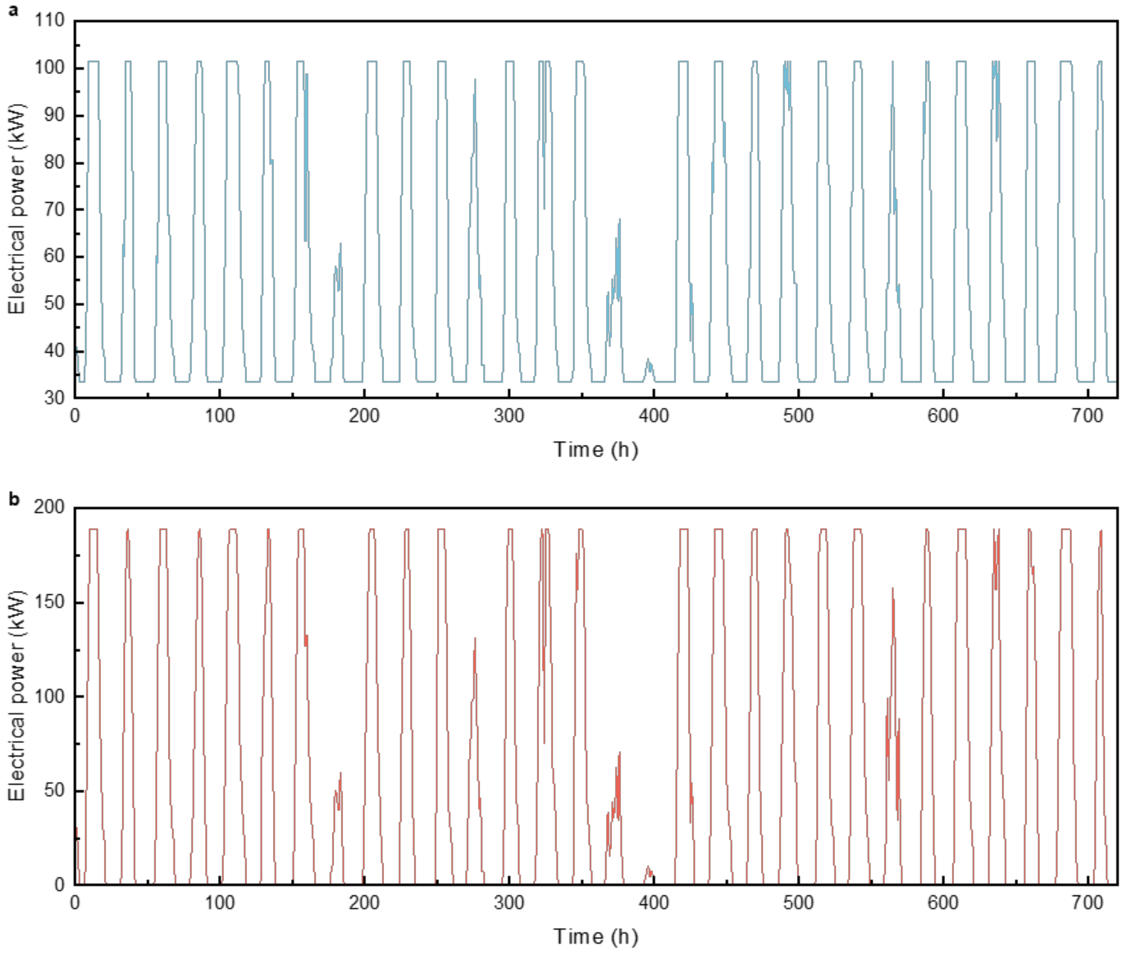

Supplementary Figure 2. Hourly electric power of electrolyzer in a typical month. a and b are WE1 and WE2, respectively.

The degradation rate of the electrolyzer (D_{EL}) is calculated as follows [15].

$$D_{EL} = \frac{H_{sto,EL} + \frac{1}{h} \times \sum_{t=1}^{t=h} \left(\frac{EP_{EL}(t)}{IC_{EL}} \times (\sigma_{EL} - H_{sto,EL}) + k_{EL} f_{EL}(t)^2 \right)}{N_{EL}} \tag{41}$$

$$f_{EL}(t) = \frac{|EP_{EL}(t) - EP_{EL}(t-1)|}{IC_{EL}} \tag{42}$$

where, EP_{EL} is the electric power of the electrolyzer, kW. N_{EL} is the lifetime of the electrolyzer, year. $H_{sto,EL}$, σ_{EL}, k_{EL} are empirical coefficients related to the degradation rate, the proton exchange membrane (PEM) electrolyzer is taken as 0.35, 0.5, 1×10^{-5}, and the alkaline (ALK) electrolyzer is taken as 2, 10, 0.1, respectively. f_{EL} is the power volatility of the electrolyzer.

Supplementary Table 8. Full load operation time and degradation rate of four water electrolysis hydrogen production systems.

System	WE1	WE2	WE3	WE4
Full load operation hours (h)	8760	8760	1679	1107
Full load operation rate (%)	100	100	19.2	12.6
Degradation rate (%)	2.5	2.5	2.2	1.9

Supplementary Table 9. Comparison of electrolyzer degradation rates between constant power operation and dynamic operation. Taking WE4 as an example, the hydrogen production rate of the electrolyzer is 1kg/h in constant power operation.

System	Constant power operation	Dynamic operation
PEM electrolyzer (%)	1.9	1.9
ALK electrolyzer (%)	14.3	15.9

Supplementary Note 4. The setting and optimization results of time-of-use electricity price

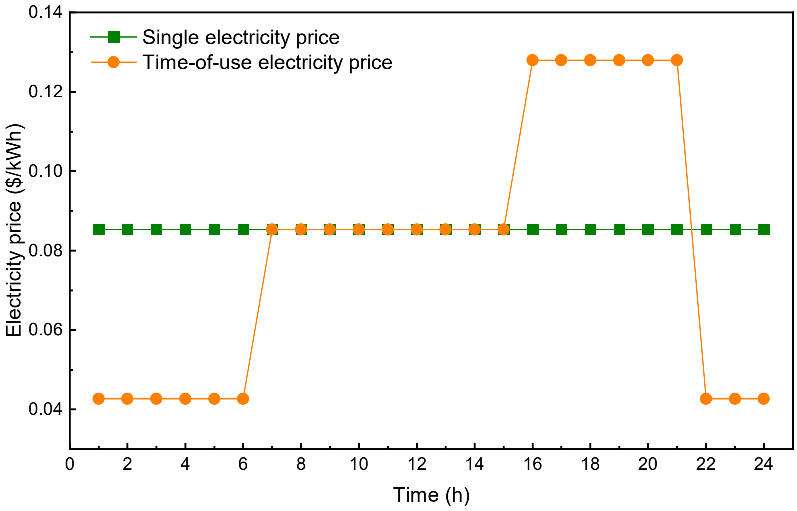

Supplementary Figure 3. Single electricity price and time-of-use electricity price, taking Beijing as an example. On the basis of the single electricity price in each province, it fluctuates by 50% as the time-of-use electricity price in each province

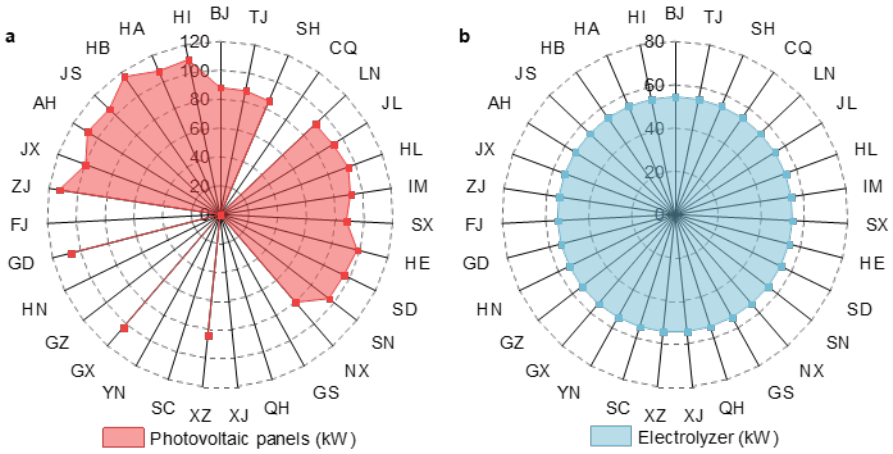

Supplementary Figure 4. Optimal configuration of WE2 system under time-of-use electricity price. a and b are the optimal capacities of the photovoltaic panels and the electrolyzer, respectively.

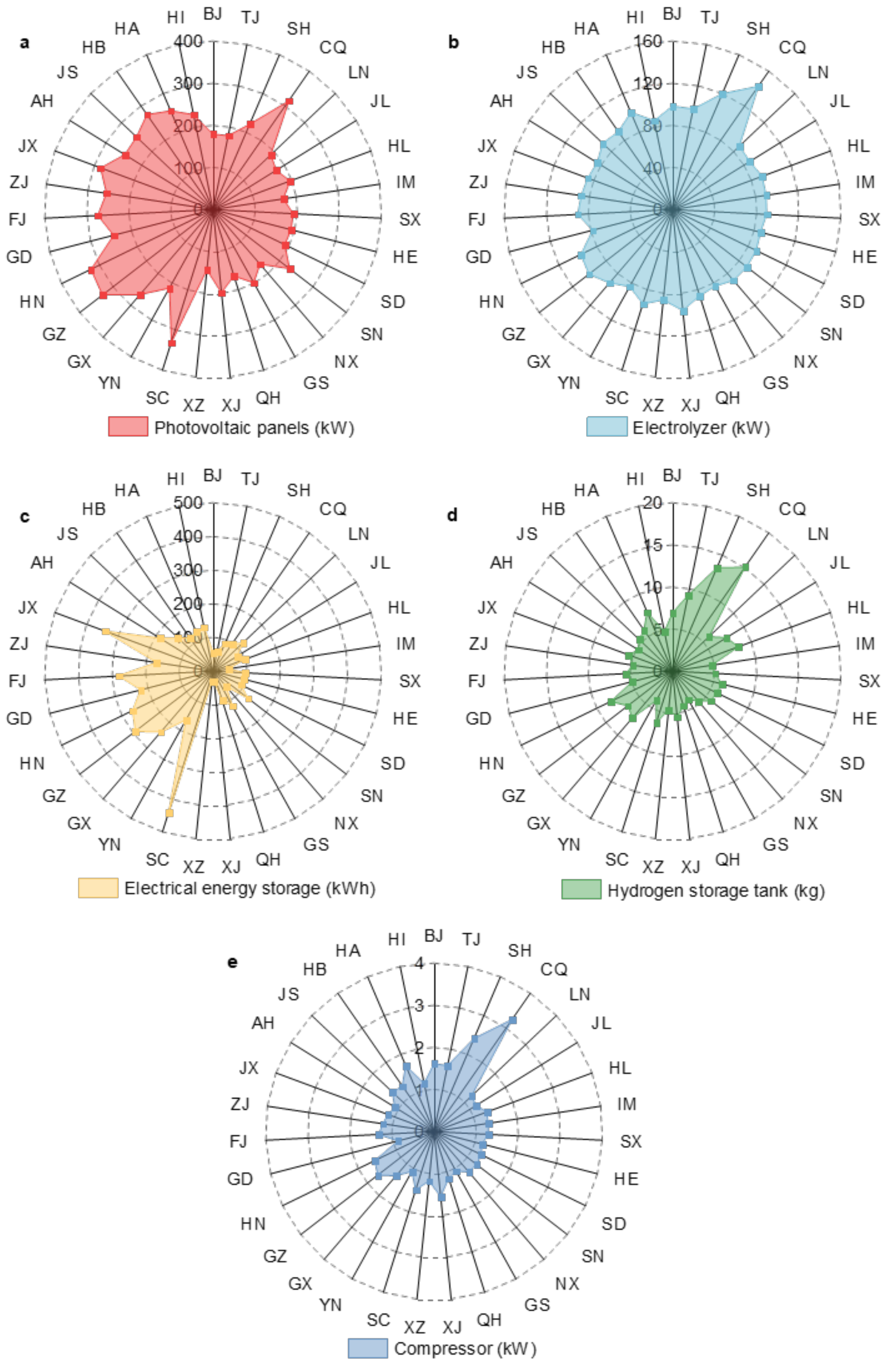

Supplementary Figure 5. Optimal configuration of WE3 system under time-of-use electricity price. a, b, c, d, and e are the optimal capacities of the photovoltaic panels, electrolyzer, electrical energy storage, hydrogen storage tank, and compressor, respectively.

Supplementary Note 5. Distribution of hydrogen demand by province

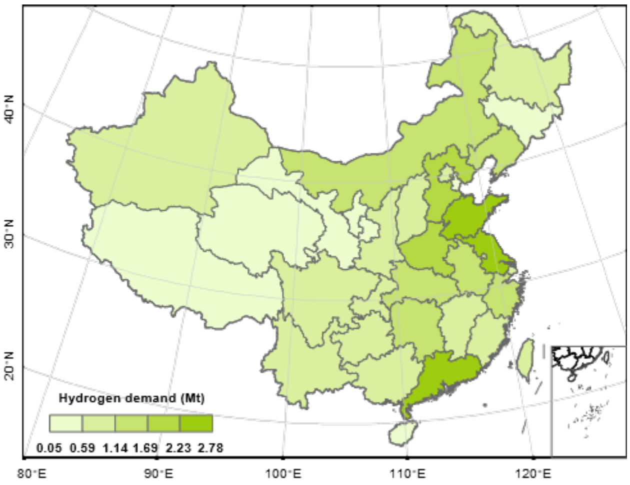

Supplementary Figure 6. Distribution of hydrogen demand by province. Map Source: Standard Map Service of the Ministry of Natural Resources, People's Republic of China [Map Review Number: GS (2020) 4619].

Supplementary Note 6. Setting parameters for the hydrogen production pathway for 2025-2050

Supplementary Table 10. Assumptions on economic and technical parameter of water electrolysis hydrogen production.

Years	2025	2030	2035	2040	2045	2050	Source
Unit investment cost of PV ($/kW)	435	386	317	298	280	262	1
Unit investment cost of electrolyzer ($/kW)	1140	855	689	523	356	190	2
Unit investment cost of electricity energy storage ($/kWh)	282	246	209	172	135	98	4
Unit investment cost of hydrogen storage tank ($/kg)	620	492	416	342	269	197	6
Unit investment cost of compressor ($/kW)	2025	1606	1359	1116	877	640	6
Conversion efficiency of electrolyzer (%)	64	69	71.50	74	78.5	83	2

Supplementary Table 11. Assumptions on carbon price and coal price.

Years	Carbon price ($/t)	Coal price ($/t)
2025	10.3	90.2
2030	16.5	93.8
2035	22.7	97.2
2040	28.9	100.7
2045	35.1	104.3
2050	41.3	107.7

Source: State Grid Energy Research Institution [16]

Supplementary Table 12. Assumptions on carbon emission factors of power grids in each province.

Province	2020	2025	2030	2035	2040	2045	2050
Beijing (BJ)	0.615	0.595	0.519	0.289	0.187	0.153	0.145
Tianjin (TJ)	0.841	0.688	0.536	0.418	0.344	0.314	0.306
Shanghai (SH)	0.548	0.333	0.325	0.281	0.251	0.237	0.233
Chongqing (CQ)	0.432	0.363	0.256	0.179	0.136	0.120	0.115
Liaoning (LN)	0.91	0.578	0.496	0.371	0.296	0.266	0.258
Jilin (JL)	0.839	0.564	0.43	0.216	0.130	0.104	0.098
Heilongjiang (HL)	0.814	0.654	0.599	0.504	0.440	0.412	0.404
Inner Mongolia (IM)	1.000	0.8	0.792	0.673	0.592	0.557	0.546
Shanxi (SX)	0.841	0.707	0.7	0.598	0.528	0.497	0.489
Hebei (HE)	1.092	0.736	0.683	0.544	0.455	0.418	0.408

Shandong (SD)	0.742	0.546	0.498	0.383	0.312	0.283	0.276
Shaanxi (SN)	0.641	0.607	0.601	0.515	0.456	0.430	0.423
Ningxia (NX)	0.872	0.724	0.665	0.459	0.345	0.302	0.291
Gansu (GS)	0.46	0.443	0.407	0.279	0.209	0.183	0.176
Qinghai (QH)	0.095	0.067	0.032	0.01	0.005	0.003	0.003
Xinjiang (XJ)	0.749	0.720	0.713	0.573	0.483	0.445	0.435
Tibet (XZ)	0.095	0.067	0.032	0.01	0.005	0.003	0.003
Sichuan (SC)	0.117	0.104	0.075	0.04	0.025	0.020	0.019
Yunnan (YN)	0.146	0.1	0.062	0.025	0.013	0.010	0.009
Guangxi (GX)	0.526	0.336	0.334	0.279	0.242	0.226	0.222
Guizhou (GZ)	0.42	0.398	0.276	0.204	0.161	0.145	0.140
Hunan (HN)	0.487	0.453	0.409	0.312	0.253	0.229	0.222
Guangdong (GD)	0.445	0.369	0.332	0.276	0.239	0.223	0.218
Fujian (FJ)	0.489	0.363	0.33	0.27	0.231	0.214	0.209
Zhejiang (ZJ)	0.532	0.418	0.386	0.307	0.257	0.236	0.230
Jiangxi (JX)	0.616	0.474	0.436	0.354	0.301	0.278	0.272
Anhui (AH)	0.763	0.755	0.694	0.596	0.529	0.499	0.490
Jiangsu (JS)	0.695	0.601	0.512	0.411	0.346	0.319	0.311
Hubei (HB)	0.316	0.31	0.254	0.202	0.169	0.155	0.151
Henan (HA)	0.738	0.599	0.49	0.389	0.325	0.298	0.291
Hainan (HI)	0.459	0.326	0.224	0.115	0.070	0.057	0.053

Source: China regional power grids carbon dioxide emission factors (2023) [14]

Supplementary Note 7. Sensitivity analysis of hydrogen supply rate

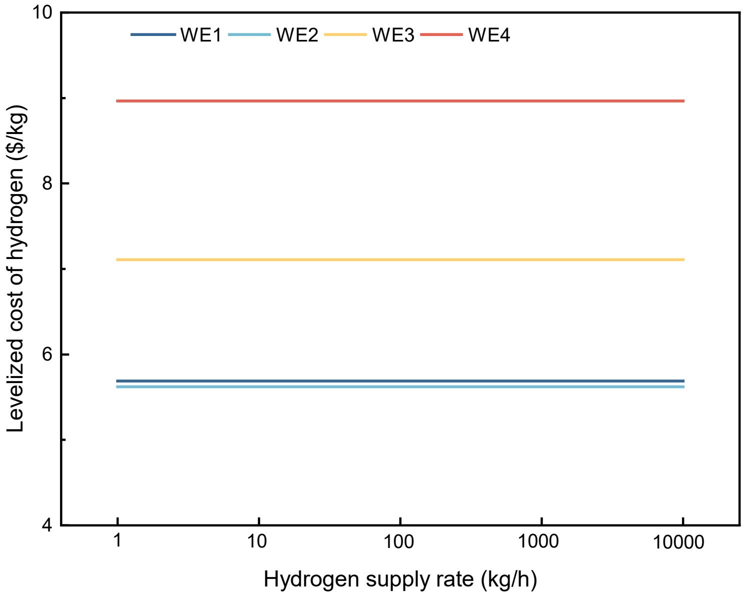

Supplementary Figure 7. Sensitivity analysis of hydrogen supply rate.

References

1. *China PV Industry Development Roadmap, 2022-2023*. China Photovoltaic Industry Association (2023).

2. Pan G, Gu W, Hu Q, Wang J, Teng F, Strbac G. Cost and low-carbon competitiveness of electrolytic hydrogen in China. *Energy & Environmental Science* **14**, 4868-4881 (2021).

3. *Comparison of Commercial, State-of-the-Art, FossilBased Hydrogen Production Technologies*. National Energy Technology Laboratory, https://www.osti.gov/servlets/purl/1862910/ (2022).

4. Bracci JM, Sherwin ED, Boness NL, Brandt AR. A cost comparison of various hourly-reliable and net-zero hydrogen production pathways in the United States. *Nature Communications* **14**, 7391 (2023).

5. Fan G*, et al.* Energy management strategies and multi-objective optimization of a near-zero energy community energy supply system combined with hybrid energy storage. *Sustainable Cities and Society* **83**, 103970 (2022).

6. Terlouw T, Bauer C, McKenna R, Mazzotti M. Large-scale hydrogen production via water electrolysis: a techno-economic and environmental assessment. *Energy & Environmental Science* **15**, 3583-3602 (2022).

7. Fan G*, et al.* Two-layer collaborative optimization for a renewable energy system combining electricity storage, hydrogen storage, and heat storage. *Energy* **259**, 125047 (2022).

8. Petkov I, Gabrielli P. Power-to-hydrogen as seasonal energy storage: an uncertainty analysis for optimal design of low-carbon multi-energy systems. *Applied Energy* **274**, 115197 (2020).

9. Guerra OJ, Eichman J, Kurtz J, Hodge B-M. Cost Competitiveness of Electrolytic Hydrogen. *Joule* **3**, 2425-2443 (2019).

10. *PVWatts Calculator*. National Renewable Energy Laboratory. https://pvwatts.nrel.gov/.

11. *Low-carbon development of hydrogen production from coal in the context of carbon neutrality* Centre for Energy and Environmental Policy Studies, Beijing Institute of Technology, https://ceep.bit.edu.cn/docs//2021-01/3e99083030724e6792f7695c1b7e2d49.pdf (2021).

12. *China Gas Prices : 36 cities*. https://www.ceicdata.com.cn/zh-hans/china/gas-

price-36-city (2024).

13. *Hydrogen Energy Industry Development Report of China 2020*. China EV100, https://sdtzsb.com/Uploads/editor/6095e307b4c84.pdf (2020).

14. *China regional power grids carbon dioxide emission factors (2023)*. Chinese Academy of Environmental Planning (2023).

15. Yu B, Fan G, Sun K, Chen J, Sun B, Tian P. Adaptive energy optimization strategy of island renewable power-to-hydrogen system with hybrid electrolyzers structure. *Energy* **301**, 131508 (2024).

16. *China Energy & Electricity Outlook 2019*. State Grid Energy Research Institution (2019).
