# Supplementary material for: Economic and environmental competitiveness of multiple hydrogen production pathways in China
Source: Nat Commun. 2025 May 8;16:4284. doi: 10.1038/s41467-025-59412-y (PMC12062354; doi:10.1038/s41467-025-59412-y)
Supplement: Supplementary file 2 — Description of Additional Supplementary Information [file 41467_2025_59412_MOESM2_ESM.docx]

**Supplementary software file legend**

Optimization model code for the hydrogen production system with fixed renewable energy penetration.
